# Correlation between interleukin-28 gene polymorphism with interleukin-28 cytokine levels and viral genotypes among HCV patients in Yazd, Iran

**DOI:** 10.1186/s13104-019-4651-z

**Published:** 2019-09-24

**Authors:** Mansour Moghimi, Forough Tavakoli, Masoud Doosti, Abbas Ahmadi-Vasmehjani, Mohsen Akhondi-Meybodi

**Affiliations:** 1 Department of Pathology, School of Medicine, Shahid Sadoughi University of Medical Sciences, Yazd, Iran; 2 Virology Department, School of Public Health, Tehran University of Medical Sciences, Tehran, Iran; 3 Infectious and Tropical Diseases Research Center, Shahid Sadoughi University of Medical Sciences, Yazd, Iran; 4 Department of Medical Virology, Faculty of Medical Sciences, Tarbiat Modares University (TMU), Tehran, Iran; 5 Gastroentrology Department, Shahid Sadoughi Hospital, Faculty of Medicine, Shahid Sadoughi University of Medical Sciences, Yazd, Iran

**Keywords:** Polymorphism, Hepatitis C, IL-28B genotype, Yazd

## Abstract

**Objectives:**

Recent studies using genome-wide association studies (GWAS) have shown the strong association between polymorphisms near the interleukin-28B (IL-28B) gene and spontaneous clearance of hepatitis C virus (HCV). The present study was designed to evaluate the association of interleukin-28 gene polymorphism with interleukin-28 cytokine levels in different viral genotypes among HCV patients in Yazd, Iran.

**Result:**

The most prevalent genotype in chronic cases was genotype 3a, and the lowest one was 2/3a. There were statistically significant differences in genotype frequency between the two studied groups for IL-28B rs12979860C/T. The frequency of CC genotype of IL-28B at rs12979860 SNP was higher in spontaneously cleared patients in comparison with chronic HCV patients. Significant association was found when serum levels of IL28B were compared to various IL-28 genotypes. There was a significant difference between IL-28 polymorphism and HCV genotypes (p = 0.003).

## Introduction

Hepatitis C virus (HCV) accounts as one of the major public health concerns [1, 2]. More than 185 million infections have estimated globally [3]. Of these, 170 million infection leads to chronic infection, resulting in 350,000 deaths annually due to cirrhosis and liver cancer [3]. The overall seroprevalence rate of HCV infection in Iran is about 0.6% [4]. A few patients (20–30%) recover spontaneously and the rest (70–80%) progress in chronic infection, of whom, 25% may develop cirrhosis and hepatocellular carcinoma [5, 6].

The hepatitis C virus has seven genotypes, which include more than 65 subtypes [7, 8]. HCV genotype 1 is the most common all around the world [9–11]. The most frequent genotype in Iran is 1 a, 3a and 1 b respectively [9]. Up until recently, pegylated interferon-alpha (PEG-IFN*α*) in combination with ribavirin was the only treatment option with limited efficacy of achieving a sustained virological response [12]. Direct-acting antivirals have recently improved the treatment of chronic hepatitis C by achieving a sustained virological response in more than 95% of hepatitis C cases [13]. But the high cost of these drugs and their serious side effects restrict their usage [14]. Many variables such as viral, host, and environmental factors may dramatically affect the outcome of HCV infection, including chronic infection or spontaneous clearance [15–17].

Recent evidence highlights that host factors may involve in the consequence of antiviral therapy [18]. IL-28A and IL-28B, two cytokine isoforms known as interferon-λ1 (IFN-λ1) and IFN-λ2 encoded by IL28B gene, which has an important role in the immune response to HCV [19, 20].

Three major studies using genome-wide association studies (GWAS), have shown the strong association between single nucleotide polymorphisms (SNPs) in the vicinity of IL28B gene, spontaneous clearance and sustained viral response (SVR) or non- response to PEG-IFNα/RBV therapy [21–23]. In fact, the mentioned polymorphisms may predict the response outcome to PEG-IFNα/RBV therapy and viral clearance [24]. The several studies have shown that HCV patients with the IL-28B-CC genotype have stronger immune protection against HCV than patients with the TT or CT genotypes [4, 12, 24, 25]. In other words, spontaneous clearance of infection is more likely in patients with genotype CC. Moreover, these patients respond to Ribavirin and Peg-Intron (peginterferon) better than patients with other genotypes.

Until recently, little is known about the association of IL28B serum levels with the different IL28B genotypes in HCV patients with different virus genotypes [12, 26]. Therefore, this study aims to evaluate the association of interleukin-28 gene polymorphism with interleukin-28 cytokine levels in different viral genotypes among HCV patients in Yazd, Iran.

## Main text

### Methods and materials

#### Patients

A cross-sectional study was done on 110 adult patients with chronic HCV infection and 65 participants who had spontaneously a resolved HCV infection referred to Shahid Sadoughi Hospital, Yazd, in 2013. Inclusion criteria for patients groups were based on positive results for anti–HCV antibody ELISA and medical records. Participants had spontaneously a resolved HCV infection verified based on laboratory tests. The study was approved by the Ethics committee of Shahid Sadoughi University of Medical Sciences-Yazd, and written informed consent was signed by each participant prior to enrollment in the study.

#### Sample collection

About 3 mL of peripheral blood was drawn from each participant and were immediately placed in sterile EDTA-anticoagulant tubes. Plasma was separated by centrifuging at 3000 rpm, for 15 min and stored at − 80 °C for later steps.

#### cDNA synthesis and subgenotyping of HCV

HCV RNA was extracted from 100 μl of patient’s sera using High Pure RNA Isolation Kit (Roche Applied. Sciences, Germany) following the manufacturer’s instruction. Extracted RNA was converted to cDNA using the RevertAid First Strand cDNA synthesis kit (Thermo Scientific; USA). The HCV genotyping was verified by type-specific primers using the AmpliSens kit (Russia) and sequencing.

#### DNA extraction

Human genomic DNA was extracted from 300 μl of the buffy coat using the AmpliSens^®^ RIBO-prep Nucleic acid extraction kit (Russia) according to manufacturer’s recommendation and was stored at − 80 °C.

#### IL-28B genotyping by PCR–RFLP and sequencing

Polymorphism at position rs12979860C/T was identified using polymerase chain reaction-restriction fragment length polymorphisms (PCR–RFLP) as previously described [27]. The PCR reaction was performed in a total volume of 25, containing 12.5 µ master mix, 0.5 µ of each specific primer, including forward: 5′-GCTTATCGCATACGGCTAGG-3′, and reverse: 5′-AGGCTCAGGGTCAATCACAG-3′, 6.5 µ DEPC, and 5 µ of the template. Then, PCR products (242 bp) were introduced into Bsh1236I (BstUI) enzyme digestion to find polymorphisms at rs12979860. The products were run on 2% agarose gel for developing the digestion pattern of each reaction. In the case of rs12979860 polymorphism, the presence of 160, 135, 82 and 25 bp bands were indicative of heterogeneous CT genotype. The appearance of 135, 82 and 25 bp bands were indicative of CC, while presences of 160 and 82 bp bands were demonstrative of homozygous status for the TT genotype. In addition, the sequencing was performed on PCR products using BigDye^®^ Terminator V3.1 Cycle Sequencing Kits (Applied Biosystems, Foster City, CA, USA) in a ABI 3730xl sequencer (Applied Biosystems, Foster City, CA, USA) according to the manufacturer’s instructions. Sequencing data were analyzed using the Chromas Lite software version 2.01 (Technelysium Pty Ltd, Australia).

#### Serum IL-28B detection by ELISA

IL-28B concentration was measured in patients’ sera using a sandwich ELISA according to the manufacturer’s instruction kit (Padgin Teb Co, Iran). This kit is used a specific antibody that has no detectable cross-reactivity with other relevant proteins.

#### Statistical method

Statistical analyses were done using SPSS v. 22.0. A Chi square test was used to investigate the genotypic distribution of IL28 SNPs among chronic hepatitis C patients, and those had spontaneously resolved HCV infection. The association between IL28B polymorphisms with IL28 serum level and age evaluated using ANOVA test. In order to explain the relationship between IL28B Polymorphisms and independent variables, logistic regression was performed. A *p* value less than 0.05 was considered significant.

### Result

In the present study, blood samples from 175 participants infected with HCV were collected. Of them, 125 participants (71.4%) were male and 50 participants (28.6%) were female. The most prevalent genotype in chronic cases was genotype 3a (60.6%), and the lowest one was 2/3a (1.1%) in our population (Table 1).

**Table 1:** HCV genotypes and distribution of IL-28 genotypes in patients with chronic HCV and spontaneous clearance

Variable	Chronic HCV	Spontaneous clearance	P value
HCV genotypes			
3a	60.6%		
1a	34.9%		
Mixed genotypes	4.5%		
rs12979860 genotypes			< 0.0001
CC	22 (33.84%)	41 (65.1%)	
CT	74 (76.28%)	23 (23.71%)	
TT	14 (93.33%)	1 (6.66%)	

#### Association of IL28B polymorphisms with HCV infection

The distribution of cytokine genotype of rs12979860C/T SNPs in chronic patients and those with cleared infection is demonstrated in Table 1. There were statistically significant differences in genotype frequency between the two studied groups for IL-28B rs12979860C/T. The frequency of CC genotype of IL-28B at rs12979860 SNP was higher in spontaneously cleared patients in comparison with chronic HCV patients (65.1% vs 34.92%) (p < 0.05). The frequency of CT haplotype was significantly higher in the chronic group than in those who spontaneously clear the infection (p < 0.05).

#### Association of IL28B polymorphisms with HCV genotypes

Based on the Chi square test, there was a significant difference between IL-28 polymorphism and HCV genotypes (p = 0.003). The CC genotype of IL-28 polymorphism shows the most prevalence in both genotypes 3a and 1a (47.6%). Also the distribution of IL-28B rs12979860 CT genotype in HCV genotype 3a was higher than HCV genotype 1a among chronic patients (72.2% vs. 24.7 percentage) and spontaneously clearance patients (72.2% vs 23.71%). In addition, distribution of IL-28B rs12979860 TT genotypes in HCV genotype 1a infected patients was 6.7% higher than HCV genotype 3a infected patients, and this rate was 40.04% higher than spontaneously clearance individuals (Table 2). The different genotypes of IL-28 are not significant based on the sex and age group (data not shown).

**Table 2:** Comparison of distribution of IL-28B genotypes and alleles respected to HCV genotypes

Variables	GT3, n = 106	GT1, n = 61	MGT, n = 8	P
rs12979860 genotypes				0.003
CC	30 (47.6%)	30 (47.6%)	3 (4.8%)	
CT	70 (72.2%)	24 (24.7%)	3 (3.09%)	
TT	6 (40%)	7 (46.7%)	2 (13.33%)	

#### Correlation between IL28B Serum Level and HCV Infection

Serum levels of IL-28 have been measured in the blood of all patients and are summarized in Table 3. The CT genotype of IL-28 shows the highest serum level (55.4%) compared to other genotypes in chronic hepatitis patients (p = 0.003). The mean level of interleukin-28 in the participants with chronic and spontaneously clearance was 119.83 ± 52.36 pg/mL and 110.34 ± 48.8 pg/mL respectively, that did not show a significant difference (p = 0.237). In accordance, linear regression which has been analyzed based on the IL-28 gene polymorphisms, demonstrated the association of rs12979860 SNP of IL28B with clearance/chronicity of HCV infection (p < 0.0001).

**Table 3:** Interleukin 28 serum levels among patients with different genotypes of IL28

IL-28 genotypes	Number (%)	IL-28 level (Mean ± SD pg/mL)	P value
CC	63 (36)	101.62 ± 43.68	0.003
CT	97 (55.4)	128.11 ± 54.88	
TT	15 (8.6)	101.6 ± 35.5	
Total	175 (100)	116.3 ± 51.13	

### Discussion

Not only the therapeutic response is variable among hepatitis C patients, but also it is difficult for some patients to tolerate the treatment. Therefore, there is a need for attention to some factors that can affect the outcome of the disease and treatment. If the prediction of disease progression, treatment response and the assessment of the risks and benefits of such therapy are possible, more logical decisions will be made on the treatment of these patients.

The distribution of rs12979860 genotypes is varied in different population. Previous studies conducted on healthy peoples in Yazd, Iran, the rate of CC, CT and TT genotypes have reported about 41.6%, 41.4% and 17.3% [25]. In the current study, the prevalence of CC, CT and TT genotypes among HCV positive patients was 36%, 55.4% and 5.6%, respectively.

An association between rs12979860 SNP of IL-28B, the strongest associated single nucleotide polymorphism (SNP) in treatment response [21], and spontaneous clearance of hepatitis C have shown [24]. The Patients with CC genotype of the IL-28B gene less progress towards chronic hepatitis C [28]. In the current study, we found the significant association in the distribution of CC genotype and C allele of IL-28B gene at rs12979860 SNP of IL-28B and spontaneous hepatitis C viral clearance rates. This finding is in agreement with the findings of Thomas et al. [24] and Rauch et al. [29]. In line with the role for this polymorphism, a strong relationship exist between the highest clearance rate among C/C homozygous genotypes, an intermediate clearance rate among C/T genotypes and the lowest rate in the T/T homozygous genotypes that agree with the findings of other studies [21, 24]. It has been reported the presence of T allele strongly enhances chronic infections in Italian populations [30]. In the current study, the most prevalent IL-28B rs12979860 genotype in chronic hepatitis C patients was CT followed by CC and TT. This finding matches those observed in earlier studies in HCV infected patients in the United State, Europe, Australia and Iran [14, 29, 31, 32].

Previous studies on HCV-positive patients with genotype 1 and 4 revealed that the virus spontaneous clearance was higher in patients with CC genotype of rs12979860 than other rs12979860 genotypes [33, 34]. In the present study, among patients with cleared HCV genotype 3a, the rate of CC, CT and TT genotypes of rs12979860 were 65%, 31% and 4%, respectively. Therefore, in contrast with results of studies mentioned above, rs12979860CC is associated with clearance of HCV genotype 3a. This finding is similar to result of study conducted by Mosavi-Nasab et al. [14]. In the current study, the distribution of CC, CT and TT genotypes of rs12979860 among patients with cleared HCV genotype 1a was not significantly different.

The frequency of rs12979860 CC in patients who were infected with HCV genotypes 3a and those infected with HCV genotypes 1a is equal and higher than other genotypes. This finding does not support the previous researches demonstrating the higher frequency of CC genotype in patients infected with HCV genotypes 3a than genotypes 1 [14, 35]. However, the frequency of IL-28B genotypes according to the HCV genotypes is not in agreement with different reports.

Our study indicated that there is a significant correlation between IL-28 variants and IL-28 cytokine serum levels. These observations are consistent with the results reported by Langhans et al., who showed a correlation of serum level with the C allele of rs12979860 SNP [26]. Al-Qahtani et al. were evaluated the association between five SNPs in IL28B gene region with the level of IL28B in serum. Only Rs8099917 SNP was significantly correlated with the IL28B levels, and the other four SNPs (including rs12979860) was not show an association with the levels of IL28B.

In conclusion, rs12979860 C allele regarded as a favorable allele determining the response to hepatitis C treatment and the spontaneous clearance of the HCV. Our data confirm the role of rs12979860 SNP in the clearance of various HCV genotypes, so it can be potentially considered as a genetic marker.

## Limitations

The small sample size was a limitation for this study.

## Data Availability

No additional file is available for this study; all the data are included in the manuscript.

## Abbreviations

GWAS: genome-wide association studies
IL-28B: interleukin-28B
HCV: hepatitis C virus
SNP: single nucleotide polymorphisms
PCR: polymerase chain reaction
ELISA: enzyme-linked immunosorbent assay
